# Waterlogging increases greenhouse gas release and decreases yield in winter rapeseed (*Brassica napus* L.) seedlings

**DOI:** 10.1038/s41598-023-46156-2

**Published:** 2023-10-31

**Authors:** Linlin Li, Lang Zhang, Jianwu Tang, Hucheng Xing, Long Zhao, Hongdong Jie, Yucheng Jie

**Affiliations:** 1 College of Agronomy, Hunan Agricultural University, Changsha, 410128 Hunan People’s Republic of China; 2 State Key Laboratory of Estuarine and Coastal Research, East China Normal University, Shanghai, 200241 People’s Republic of China; 3 Hunan Provincial Key Laboratory of Crop Germplasm Innovation and Utilization, Changsha, 410128 People’s Republic of China

**Keywords:** Agroecology, Ecosystem ecology

## Abstract

A sustainable future depends on increasing agricultural carbon (C) and nitrogen (N) sequestration. Winter rapeseeds are facing severe yield loss after waterlogging due to the effects of extreme rainfall, especially in the seedling stage, where rainfall is most sensitive. Uncertainty exists over the farming greenhouse gas (GHG) release of rapeseed seedlings following the onset of waterlogging. The effect of waterlogging on GHG release and leaf gas exchange in winter rapeseed was examined in a pot experiment. The experiment included waterlogging treatments lasting 7-day and 21-day and normal irrigation as a control treatment. According to our findings, (1) The ecosystem of rapeseed seedlings released methane (CH_4_) and nitrous oxide (N_2_O) in a clear up change that was impacted by ongoing waterlogging. Among them, N_2_O release had a transient rise during the early stages under the effect of seedling fertilizer. (2) The net photosynthetic rate, transpiration rate, stomatal conductance, plant height, soil moisture, and soil oxidation–reduction potential of rapeseed all significantly decreased due to the ongoing waterlogging. However, rapeseed leaves showed a significant increase in intercellular carbon dioxide (CO_2_) concentration and leaf chlorophyll content values after waterlogging. Additionally, the findings demonstrated an extremely significant increase in the sustained-flux global warming potential of the sum CO_2_-eq of CH_4_ and N_2_O throughout the entire waterlogging stress period. Therefore, continuous waterlogging can increase C and N release from rapeseed seedlings ecosystem and decrease yield. Therefore, we suggest increasing drainage techniques to decrease the release of agricultural GHGs and promote sustainable crop production.

## Introduction

One of the significant challenges that humanity is facing is climate change. In the Paris climate agreement, the UN member states formalized the world’s commitment to reduce greenhouse gas (GHG) emissions to hold global warming to well below 2 °C and possibly below 1.5 °C in response to global warming^1^. Carbon dioxide (CO_2_), methane (CH_4_), and nitrous oxide (N_2_O) are the three gases that contribute 66, 20, and 10%, respectively, to global warming^2,3^. Since the pre-industrial era (280 ppm), atmospheric CO_2_ concentrations have increased; by 2021, they will reach 415.7 ppm^4^. It will reach 1000 ppm this century^5^. CH_4_ and N_2_O are among the most significant GHGs in the atmosphere after CO_2_ because of their significantly greater radiative forcing effects^6^.

It is known that agricultural activities account for 30% of the world’s anthropogenic GHG release^5^. Agricultural soil has been the main source of N_2_O and CH_4_ emissions, accounting for approximately 66% and 50% of total emissions, respectively^7,8^. Currently, methanogens are known to produce CH_4_ through hydrogenotrophic, acetoclastic, and methylotrophic methanogenesis^9^. Although N_2_O can be produced under both anaerobic conditions (via denitrification) and aerobic conditions (via nitrification), the majority of N_2_O production occurs in waterlogged soils^10^.

Rapeseed (*Brassica napus* L.) is one of the most significant oilseed crops in the world. Rapeseed is cultivated on 67 million hectares in China yearly, yielding 4.5 million tons of seeds^11^. The Yangtze River Basin is the largest rapeseed-producing region in China^12^. Extreme rainfall has recently emerged as an issue for rapeseed production in the Yangtze River Basin^13^. Rapeseed, particularly, experienced waterlogging at the seedling and flowering stages, which negatively impacted grain yield^14^.

The seedling stage of a plant’s life cycle is when most plants are most sensitive to environmental change. Seedlings in such environments may sustain hypoxia and anoxia damages due to a notable reduction in gas diffusion in floodwaters after waterlogging^15^. Additionally, under anaerobic conditions, fermentation converts pyruvate into lactate or ethanol, and metabolic processes like photosynthesis, respiration, and ion transport are significantly impaired, slowing growth^16–19^. Rapeseed seedlings have significantly lowered plant height, leaf area, total root length, and dry matter^14,20,21^. The overall result was a decrease in the yield of rapeseed grains^22^. Studies demonstrate that after waterlogging, rapeseed seedlings exhibited growth retardation and delayed development^23^. Additionally, Frolking, et al.^24^ have suggested that flooded rice fields release methane, while during the transition from flooded to drained state, nitrous oxide is emitted. It has been reported that adopting alternate wetting and drying (AWD) technique can effectively reduce methane emissions under field conditions in rice fields^25^. However, far less is known about GHGs releases in rapeseed ecosystems, especially when subjected to waterlogging during the seedling stage. In the seedling stage of rapeseed, we hypothesized that waterlogging could increase CH_4_ and N_2_O emissions. Our objectives were as follows: (1) to monitor the water content and redox potential of the soil in the root zone, simultaneously to measure agronomic traits such as plant height, leaf chlorophyll content (SPAD) value, and grain yield; (2) to compare leaf photosynthetic properties; (3) to evaluate the potential impacts of the CH_4_ and N_2_O emissions on global warming.

## Materials and methods

### Experimental design

The experiment was designed as follows. From November 2021 to June 2022, a pot experiment was carried out in the experimental farm of East China Normal University, Shanghai, China (N31° 02′ 10″, E121° 26′ 55″). However, abundant rainfall in the region is unevenly distributed throughout the seasons due to the subtropical monsoon climate. About 16 °C is the average annual temperature.

The soil (pH 7.3) included 18.60 g kg^−1^ of soil organic matter, 1.12 g kg^−1^ of total nitrogen, 24.64 mg kg^−1^ of available phosphorus, and 108.08 mg kg^−1^ of available potassium. A single-factor, entirely randomized design was used in this experiment to investigate the effects of various waterlogging times on rapeseed. On October 28, 2021, rapeseed seeds were sown. After 80 days from sowing (January 14, 2022), the waterlogging was conducted. In the test, three treatments—waterlogging lasting 7-day and 21-day and one control treatment (normal irrigation, CK)—was established. Hunan Agricultural University provided the rapeseed cultivar used for oilseeds, Zhongyou 821. Rows were 0.15 m apart, and plants were spaced 0.1 m apart. Seeds were manually spread by direct seeding in November, and harvested in May. Only the healthiest seedling per location was kept within 20d of germination. The pot’s dimensions were 0.635 × 0.425 × 0.400 m. Each treatment was repeated four times.

The soil sample for the test was collected from the plow layer of a rice-rapeseed rotation field at Wujing farm, Minghang, Shanghai. After the soil was air-dried, thoroughly mixed, and 5.0 mm sieved, 75 kg of soil was added to each plastic pot. Organic manure (100 g pot^−1^, November 2, 2021) was applied and homogenized by hand mixing as base fertilizers. Each pot received an equal amount (20 g) of seedling fertilizer (nitrogen: phosphorus: potassium = 15: 15: 15, January 20, 2022). The experiment was carried out in a partially controlled environment by growing the rapeseed seedling in a rainout shelter, stimulating controlled irrigation, and neglecting the impact of unpredictable rainfall. A consistent water level of at least 3 cm was maintained throughout the waterlogging, which lasted for 7d or 21d. After the waterlogging treatment, the water in the pot was released, restoring normal water management when soil moisture in the plastic basin reached 20–30% of the field’s water capacity. The other cultivation and management measures were the same as the regular field management measures, except for water control.

### Sampling and analytical methods

*Collecting plant samples* Plant height and SPAD (A portable chlorophyll meter, SPAD 502 Plus, Konica Minolta Optics, Japan) were measured on the 5th, 21st, and 42nd days Midway through May, when the seeds reached maturity (mid-May), they were harvested from each pot and sun-dried to a set weight.

*Key environmental parameters* After field sampling, the soil moisture was measured by Decagon EC‐5 sensors (Decagon Devices, Pullman, WA, USA). An oxidation–reduction potential (ORP) meter (FJA‐6, Nanjing, China) was used to measure soil ORP.

*Leaf gas exchange measurements* The Li-6800R portable photosynthesis system (Lincoln, NE, USA) was used to measure the leaf gas exchange measurements of net photosynthetic rate (*A*, µmol m^−2^ s^−1^), stomatal conductance (*Gsw*, mol m^−2^ s^−1^), intercellular CO_2_ concentration (*Ci*, mol m^−2^ s^−1^), and transpiration rate (*E*, mol m^−2^ s^−1^). The following are the precise operations: The light-saturated photosynthetic photon flux was 1200 μmol m^−2^ s^−1^, the reference CO_2_ concentration was 400 μmol mol^−1^, and the airflow rate into the leaf cuvette was 500 mL min^−1^. The temperature (15℃) and humidity (60%) were kept constant. On February 26, 2022 (42nd days after exposure to water stress), measurements were taken from 8:30 to 11:30 AM. Each seedling was periodically sampled over time, with the second or third most recently matured leaf taken from the apical meristem. Each treatment was replicated four times for a total of 20 leaves per pot.

*Collecting gas samples* In situ CO_2_, CH_4,_ and N_2_O fluxes were monitored with a static chamber during the waterlogging using a high-precision GHG analyzer (CO_2_/CH_4_/N_2_O/H_2_O Analyzer; Picarro-G2508, Picarro Inc., USA). Poly methyl methacrylate (PMMA) was used to create the static chamber with a thickness of 0.30 cm. The chamber measured 0.2 m in length, 0.15 m in width, and 0.45 m in height. The gas samples were collected in real-time on 5th (January 19, 2022), 7th (January 21, 2022), 10th (January 24, 2022), 16th (January 30, 2022), and 42nd (February 26, 2022) days after exposure to water stress. All gas samples were collected in the field from 9:00 to 11:00 AM. In addition, within a sampling date, the gas sampling time interval was 5 min and the measurement frequency was 1 Hz.

### GHG fluxes and sustained‐flux global warming potential calculations

GHG fluxes were calculated using the following formula:

1 $$F=\frac{dc}{dt}\times \frac{PV}{RAT}$$

where *F* is the GHG flux (μmol·m^−2^·s^−1^); dc/dt is the rate of change of GHG concentration (ppm) with time t (s); P is the air pressure, the standard is 101.2237 × 10^3^ (Pa); V is the effective volume (m^3^) of the static closed chamber; R is the gas constant, defaulted to 8.3144 (J mol^−1^ K^−1^); A is the chamber coverage (m^2^); and T is the average soil temperature (T = 273.15 °C)^26^.

This study estimated the dynamics of total radioactive forcing using the sustained-flux global warming potential (SGWP). The total emission of CH_4_ and N_2_O was calculated in mass CO_2_ equivalents (CH_4_: 45; N_2_O: 270) over a time horizon of 100 years^27^. We only calculated daytime (8–18 h) SGWP scaled to a day due to a lack of nighttime flux measurement. The following formula was used to determine how much CH_4_ and N_2_O emissions contribute to SGWP:

2 $$S{GWP}_{Ratio}=\frac{{SGWP}_{(CH4+N2O)}}{SGWP}=\frac{(45\times {F}_{CH4})+(270\times {F}_{N2O})}{\left[{F}_{CO2}+\left(45\times {F}_{CH4}\right)+\left(270\times {F}_{N2O}\right)\right]}$$

where *F*_CO2_, *F*_CH4_, and *F*_N2O_ are mass flux in units (e.g., μg CO_2_ m^−2^ s^−1^); SGWP _(CH4+N2O)_, expressed as CO_2_ equivalents, are 45 and 270, respectively, multiplied by their respective flux values (μg CO_2_ m^−2^ s^−1^); SGWP represents the total greenhouse gas warming potential expressed as CO_2_ equivalents (μg CO_2_ m^−2^ s^−1^). The *SGWP*_ratio_ is the ratio of *SGWP*_(CH4+N2O)_ to the total *SGWP*.

### Statistical analysis

Microsoft Excel 2018 for Windows was used to tabulate the data. Data analysis was performed using SPSS (IBM SPSS 23.0, SPSS Inc). One-way analysis of variance (ANOVA) was used to determine significant intergroup differences of each parameter. GraphPad Prism 8 software was used to create the graphics.

## Results

### Effects of waterlogging stress on plant and soil characteristics in rapeseed seedlings

**Figure 1:**
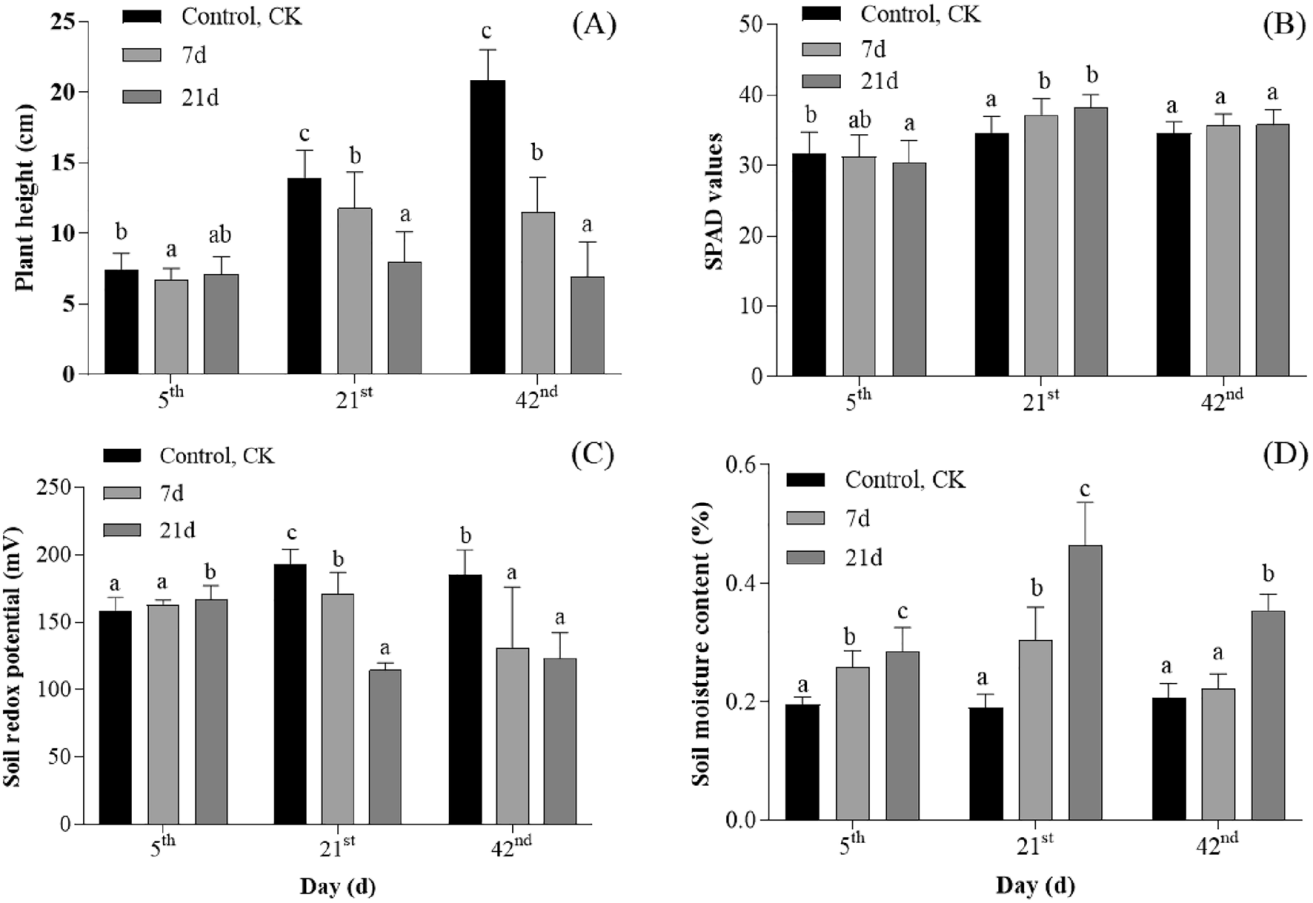
Effects of waterlogging stress on plant and soil characteristics in rapeseed seedlings. Treatment consisted of one control treatment, normal irrigation (control, CK), and two treatments: waterlogging lasting 7-day and 21-day. Significant differences among sets at the maximum points are denoted by lowercase letters above each bar (*P* < 0.05). (**A**) Represent plant height, (**B**) represent SPAD values, (**C**) represent soil redox potential, (**D**) represent soil moisture. The x-axis denotes the duration in days post-waterlogging exposure. Bars and error bars represent mean and standard error.

The variation in SPAD, plant height, soil moisture, and ORP values during waterlogging is depicted in Fig. 1. Plant height revealed growth retardation as waterlogging stress treatment time increased. Compared to CK, the waterlogged plant’s height was significantly lowered (*P* < 0.05, Fig. 1A) after waterlogging 21-day treatment. Plant height decreased significantly (*P* < 0.05, Fig. 1A) on waterlogging 7-day and 21-day treatments after waterlogging 21st days (February 4, 2022). The ORP in all the waterlogged soils exhibited similar trends, with declining values over time and lower average values in the waterlogging 21-day treatment (*P* > 0.05, Fig. 1C). The SPAD value of the leaves increased (*P* > 0.05, Fig. 1B) after waterlogging during the seedling stage. Significant differences between waterlogging 7-day (or 21-day) treatments and CK were observed in terms of the soil moisture (*P* > 0.05, Fig. 1A). After waterlogging, the soil moisture gradually reduced.

### Effects of waterlogging stress on the gas exchange in rapeseed leaves

**Figure 2:**
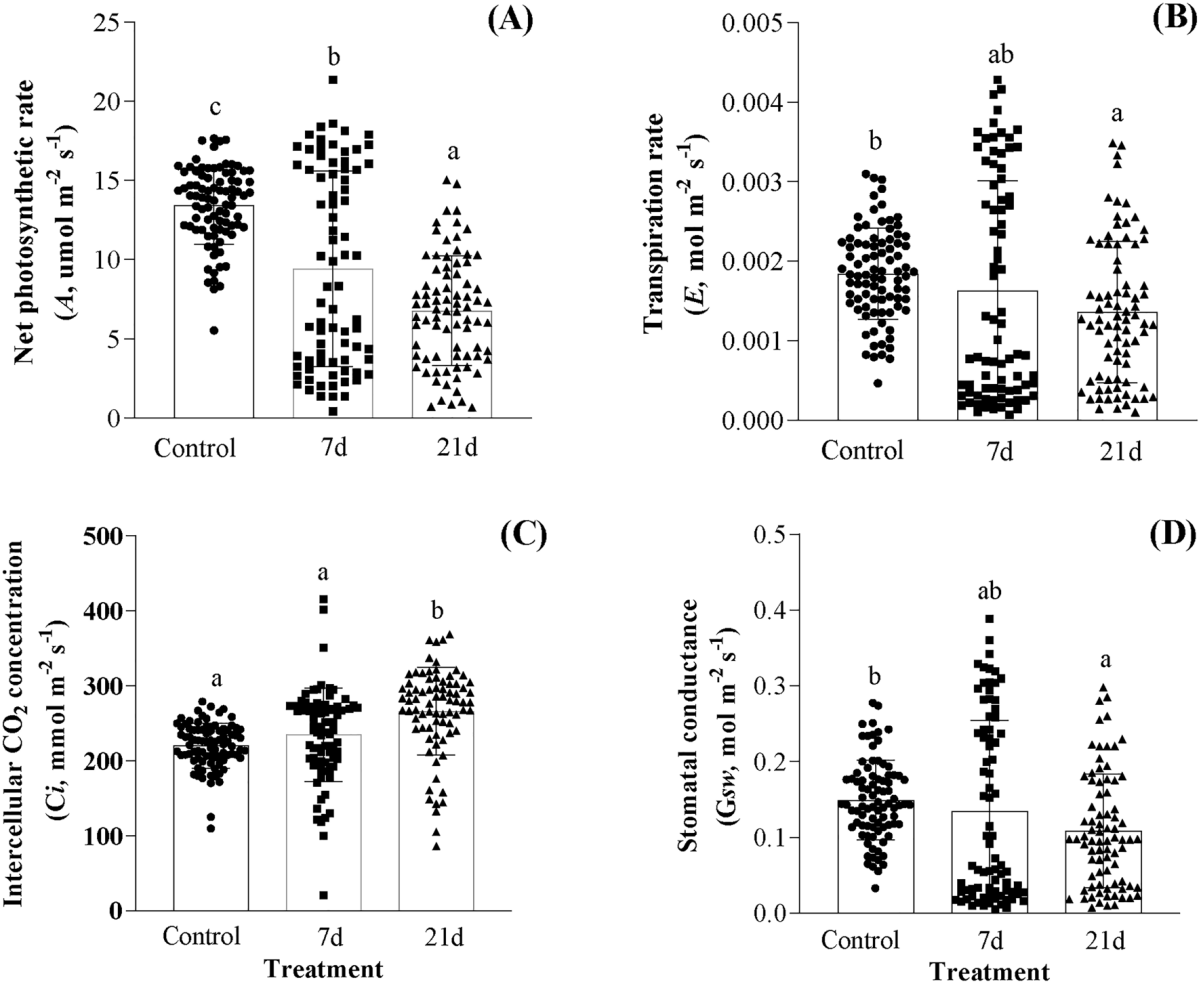
Effects of waterlogging stress on the gas exchange in rapeseed leaves. Treatment consists of one control treatment, normal irrigation (control, CK), and two treatments: waterlogging lasting 7-day and 21-day. Significant variations among sets at the maximal points are denoted by lowercase letters above each bar (*P* < 0.05). (**A**) Represent net photosynthetic rate, *A*; (**B**) represent transpiration rate, *E*; (**C**) represent intercellular CO_2_ concentration, *Ci*; (**D**) represent stomatal conductance, *Gsw*. Bars and error bars represent mean and standard error. Each black symbol (circle, square, triangle) represents a single sample of control, 7d and 21d, respectively, and the distance between the samples represents the difference in composition of the samples.

*A*, *E*, *Gsw*, and *Ci* variations during waterlogging are shown in Fig. 2. On waterlogging 7-day and 21-day treatments, *A* was significantly (*P* < 0.05, Fig. 2A) decreased compared to the control. *A* was at its lowest point at waterlogging 21-day treatment, although there was no significant difference between waterlogging 7-day and waterlogging 21-day (Fig. 2A). Furthermore, waterlogging 21-day treatment showed a significant decrease in *E* compared to the control, although waterlogging 7-day treatment showed no significant difference (*P* > 0.05, Fig. 2B) from the control, with the waterlogging 21-day treatment mark seeing a significant increase. *Ci* in the control was lower than in the 7-day and 21-day waterlogging treatments, with the waterlogging 21-day treatment mark significantly increasing (*P* > 0.05, Fig. 2C). All waterlogging treatments demonstrated the opposite tendency of the *Ci*, with a significant decrease in *Gsw* at waterlogging 21-day treatment compared to the control (*P* > 0.05, Fig. 2D).

### Effects of waterlogging stress on GHGs release in the rapeseed ecosystem

**Figure 3:**
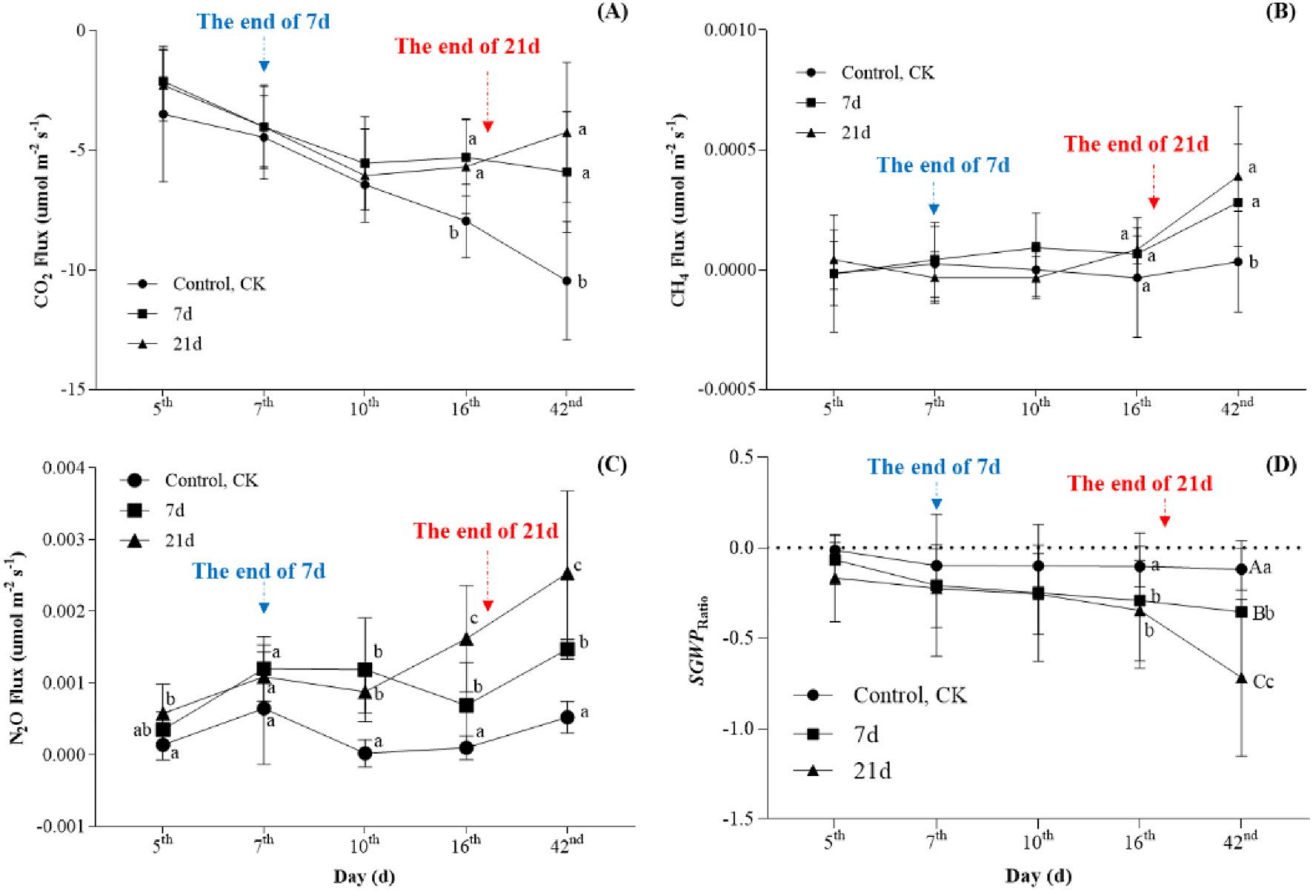
Effects of waterlogging stress on GHGs release in the rapeseed ecosystem. Treatment consists of one control treatment, normal irrigation (control, CK), and two treatments: waterlogging lasting 7-day and 21-day. Different uppercase letters represent highly significant (*P* < 0.001), different lowercase letters represent significant (*P* < 0.05), and the same letters represent no significant (*P* > 0.05). (**A**) Represent CO_2_ Flux, (**B**) represent CH_4_ Flux, (**C**) represent N_2_O Flux, (**D**) represent *SGWP*_ratio_. The x-axis denotes the duration in days post-waterlogging exposure. The end of 7d (or 21d) represents the time point when the waterlogging ends on the 7th day (or 21st day).

The effects of waterlogging on total GHG emissions are shown in Fig. 3. CO_2_ flux increased initially and then decreased as the waterlogging period increased (Fig. 3A). Throughout the waterlogging period, no statistically significant differences in CH_4_ flux were found across all groups (*P* > *0.05*, Fig. 3B). At the end of waterlogging, the CH_4_ flux was a trend for higher waterlogging 7-day and 21-day treatments, it did reach significance (*P* < 0.05, Fig. 3B). However, the rapeseed field in the waterlogging 7-day and 21-day treatments increased significantly N_2_O emissions (*P* < 0.05, Fig. 3C) compared to the control. The N_2_O fluxes of all groups had a wave change trend of initially increasing, then decreasing, and then increased during the observation period. In contrast, the overall treatments N_2_O fluxes increased after fertilization on the 6th days (January 20, 2022), and treatments for waterlogging 7-day and 21-day treatments increased more quickly than the control. Subsequently, the N_2_O fluxes of both waterlogging 7-day and 21-day treatments drastically increased compared to the control, reaching significant levels after waterlogging lasting 10th days (January 24, 2022). However, after waterlogging developed, the combined CO_2_-eq emissions from CH_4_ and N_2_O played a role in providing negative feedback. *SGWP*_ratio_ considerably decreased (*P* < 0.001, Fig. 3D) on waterlogging 7-day and waterlogging 21-day compared to the control (not significantly different from day 0d to 7d).

### Effects of waterlogging stress on yield in the rapeseed ecosystem

**Figure 4:**
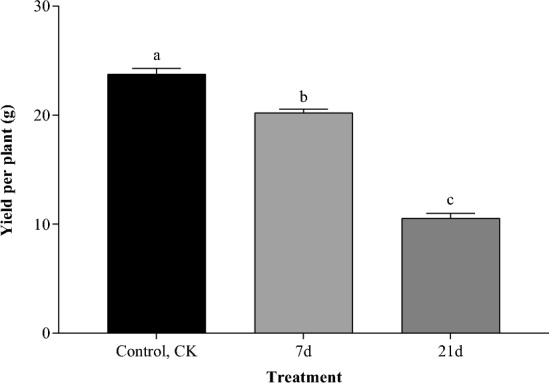
Effects of waterlogging stress on yield in the rapeseed ecosystem. Treatment consists of one control treatment, normal irrigation (control, CK), and two treatments: waterlogging lasting 7-day and 21-day. Bars and error bars represent mean and standard error. Lowercase letters above each bar indicate significant differences among sets at the maximal points (*P* < 0.05).

The yield of rapeseed variations during waterlogging are shown in Fig. 4. The yield of rapeseed significantly decreased (*P* < 0.05) after 7-day and 21-day of waterlogging when compared to the control. Compared to the waterlogging 7-day treatment, the rapeseed yield significantly decreased in the waterlogging 21-day treatment (*P* < 0.05).

## Discussion

### Effects of waterlogging stress on agronomy traits and soil characteristics in rapeseed

Crop growth and development are directly reflected in plant height and yield. According to earlier research, waterlogging significantly affected plant height^28^. In this study, shorter average plant height and decreased rapeseed yield were expected during waterlogging stress periods. Rapeseed plant heights were specifically in the range of normal irrigation > 7-day of waterlogging > 21-day of waterlogging as the waterlogging period increased (*P* < 0.05). Evidence that soil moisture increases quickly after waterlogging was obtained through field investigations (Fig. 1D). Meanwhile, waterlogging results in a lowered redox potential (Fig. 1C), which is statistically significantly different from controls (*P* < 0.05) using normal irrigation. Shabala et al.^29^ agreed with this view as well. The observed decrease in plant height and yield during the waterlogging stress periods can be attributed to several factors. Firstly, waterlogging leads to reduced oxygen availability in the root zone, resulting in hypoxic conditions for the plant roots^30^. This oxygen deficiency can negatively affect root respiration and nutrient uptake, ultimately leading to stunted plant growth^31^ and reduced crop yield. Moreover, the decrease in redox potential observed in the waterlogged conditions indicates a shift towards anaerobic conditions in the soil (Fig. 1C). Under anaerobic conditions, there is limited availability of essential nutrients such as nitrogen and phosphorus, which are crucial for plant growth and development^32^. The nutrient imbalance caused by waterlogging can further impair the overall growth and vigour of rapeseed plants.

In addition, the association between leaf chlorophyll concentration and leaf surface photosynthetic efficiency^33^. Measurements with the SPAD were used in this study to assess the relative chlorophyll content^34^. Our findings indicated that the SPAD values in the 7-day or 21-day waterlogging treatments were higher than those in the normal irrigation (Fig. 1B). However, previous studies have shown that waterlogging stress dramatically decreased the total leaf chlorophyll concentration of rapeseed seedlings^14,35^. This finding is in contrast to the results of this study. After waterlogging, an increase in SPAD values in rapeseed plants may be attributed to multiple factors. First, waterlogging restricts oxygen supply to the roots and reduces nutrient availability, resulting in hypoxia and nutrient limitation in rapeseed plants^31,36^. Under such conditions, plants may respond by enhancing chlorophyll synthesis to adapt to the changing environment, improve photosynthetic efficiency, and sustain growth vigor, thereby increasing SPAD values^37^. Second, waterlogging stress can alter leaf anatomy and cellular morphology, affecting the accumulation and distribution of chlorophyll within the leaves, consequently leading to an increase in SPAD values^17^. Studies have suggested that under waterlogging conditions, adjustments in leaf anatomy, such as increased stomatal density and reduced leaf thickness, may promote chlorophyll accumulation and enhance light energy utilization efficiency. It is important to note that while an increase in SPAD values may indicate an elevation in chlorophyll content, it does not necessarily translate to an increase in plant height in rapeseed. Other factors including stomatal limitations, nutrient imbalances, and metabolic abnormalities could also influence plant growth and yield under waterlogging stress^38^.

### Effects of waterlogging stress on the gas exchange in rapeseed leaves

Water stress is one of the main factors limiting the output of crops, which also affects plant physiology. According to Kuai, et al.^39^, under waterlogging stress, the photosynthetic leaf rate of rapeseed decreased at the seedling and bolting stages. As observed in the present study, when compared to the control, *A* was significantly decreased (*P* < 0.05) on waterlogging 7d and waterlogging 21d (Fig. 2A). According to Pandey, et al.^40^, waterlogging stress decreased *A* rate and Rubisco activity. This was accompanied by a decrease in *Gsw* (Fig. 2D), which led to less starch in the leaves. Chlorophyll concentration is a well-known key indicator of the photosynthetic ability of plants. However, in our experiment, waterlogging treatments increased the SPAD values of rapeseed leaves (Fig. 1B), which led to a decrease in the photosynthetic rate of rapeseed leaves (Fig. 2A). Further analysis of Fig. 2A revealed that the control had a more concentrated photosynthetic rate than the waterlogging treatment, which had a more scattered rate. This difference is probably caused by the various methodologies that were used. Additionally, we hypothesized that the rapeseed leaves under waterlogging stress quickly accumulated large quantities of carbohydrates and energy. Rapeseed under stress conditions require significant energy to maintain their growth and development through self-repairing behavior, but this needs further research. According to Rao, et al.^41^, the photosynthetic system of mulberry seedling leaves can self-repair in a flooded environment. This compensatory mechanism for self-regulation has been proposed by Zhang, et al.^42^. Additionally, in this study, waterlogging 21d displayed a significant decrease (*P* < 0.05) in *E* compared to the control, and waterlogging 7d displayed no significant difference (*P* > 0.05) in *E* compared to the control (Fig. 2B). Stomata are the “windows” for exchanging gases between plant leaves and their surroundings. The condition of stomatal conductance (*Gsw*) has a direct impact on the photosynthetic and transpiration ability of plants (Fig. 2D). *Gsw* decreased at 7d and 21d following waterlogging compared to the control, with a significant increase observed at 21d (*P* < 0.05). The *Ci* in all waterlogging treatments showed the opposite trend compared to the *Ci*, with a significant increase at 21d compared to the control (*P* < 0.05, Fig. 2C).

### Effects of waterlogging stress on GHGs release and yield in rapeseed ecosystem

Waterlogging is an important source of anthropogenic greenhouse gas (GHG) emissions. We previously hypothesize that waterlogging increases CH_4_ emissions while decreases N_2_O emissions. In this study, as waterlogging occurs, we observe an elevated CH_4_ emission rate in the rapeseed ecosystem, which aligns with our expected results. The primary reason for this increase is the excessive soil moisture resulting from waterlogging, creating an anaerobic environment favorable for the growth and reproduction of methanogenic bacteria, which release a significant amount of CH_4_. This finding is consistent with previous comprehensive reviews on methanogenesis and methanotrophy in soil^43^. Moreover, in this study, we also observe a significant increase in N_2_O emissions. From our analysis, we attribute this increase to the effect of soil moisture on soil N mineralization, as it partially regulates the temporal fluctuations of soil N mineralization^44,45^. Multiple studies demonstrate that water content is a crucial controlling factor influencing soil N_2_O emissions. Specifically, the amount of soil pore water content determines the magnitude of N_2_O emissions during soil rewetting^46^. Hence, under short-term waterlogging conditions, there is an increased flux of N_2_O^47^, further corroborating our research findings.

There are multiple potential causes for waterlogging in rapeseed fields, which can be broadly categorized into two main groups. One possible cause is excessive precipitation, leading to excessive soil moisture. This may be attributed to frequent or extreme rainfall events resulting from climate change^48^. Another potential cause is poor soil drainage, which hinders the rapid removal of excess water. This may be due to factors such as poor soil structure, high soil density, or inadequate surface drainage systems^49,50^. Regardless of the reasons, based on the findings of this study, whenever waterlogging occurs, the emissions of CH_4_ and N_2_O greenhouse gases in rapeseed fields increase, along with an increase in the *SGWP*_ratio_ (Fig. 3). This not only directly affects the yield of rapeseed in the current year but also exacerbates the global warming potential, subsequently impacting the next year's rapeseed production. Therefore, in addition to addressing the drainage issues in rapeseed fields, reducing greenhouse gas emissions is equally essential in addressing waterlogging problems. This series of measures contributes to the sustainable development of rapeseed production.

## Conclusions

We discovered that rapeseed exposed to waterlogging stress released more GHGs than they would under normal irrigation. When compared to normal irrigation, waterlogging for 7-day or 21-day resulted in decreased yield, plant height, soil moisture, soil ORP, *A*, *E*, and *Gsw*. Compared to normal irrigation, waterlogging for 7-day or 21-day increased SPAD values and *Ci*. The* SGWP*_ratio_ of the sum CO_2_-eq of CH_4_ and N_2_O significantly increased compared to normal irrigation during the entire flooding stress period. We concluded that continuous waterlogging over a short period could decrease rapeseed yield and increase seedling rapeseed C and N release, further contributing to global warming. Therefore, we suggest increasing drainage techniques to decrease the release of agricultural GHGs and promote sustainable crop production.

## Data Availability

The datasets used and/or analysed during the current study available from the corresponding author on reasonable request.
